# Trends of violence among 7th, 8th and 9th grade students in the state of Lara, Venezuela: The Global School Health Survey 2004 and 2008

**DOI:** 10.1186/0778-7367-69-7

**Published:** 2011-11-01

**Authors:** Ricardo Granero, Esteban S Poni, Bertha C Escobar-Poni, Judith Escobar

**Affiliations:** 1 Epidemiology Unit, ASCARDIO, Carrera 17, Barquisimeto, 3001, Venezuela; 2 The Good Samaritan Hospital, Department of Pediatrics, Section of International Research, Avenida Severiano Km 1.4, Aguadilla, 00603, Puerto Rico; 3 Department of Pathology and Human Anatomy, Loma Linda University, 24760 Stewart Street, Loma Linda, 92350, USA

**Keywords:** Adolescents, Behavior, Bullying, Health Education, Physical Fight, Robbery, Unsafe School, Venezuela, Violence, Weapon

## Abstract

**Background:**

Violence by young people is one of the most visible forms of violence and contributes greatly to the global burden of premature death, injury and disability.

**Methods:**

The Global School-based Student Health Survey (GSHS), State of Lara, Venezuela (GSHS-Lara) is a school-based surveillance system. It comprises a repeated, cross-sectional, self-administered survey drawn from a representative sample of 7th to 9th grade students, performed in the school years 2003-2004 (GSHS-Lara 2004) and 2007-2008 (GSHS-Lara 2008). It explores, among other things, a general violence indicator such as school absenteeism due to feeling unsafe at school or on the way to or from school for any reason; and more specific indicators of violence such as robbery, bullying, physical fights and use of weapons, as well as exposure to lectures on how to prevent violence. Results are given in terms of prevalence percentage.

**Results:**

Absenteeism doubled between the two study periods (10.8% to 20.8%). The number of students that were a victim of robbery remained high and without change both outside (14.2% and 14.8%) and inside school (21.7% and 22.0%). The number of victims of bullying was high and increasing (33.4% and 43.6%). Bullying associated with being physically attacked decreased (18.5% to 14.3%). Physical attacks without active participation and not associated with bullying were frequent (21.5%). Physical fighting with active participation prevalence remained high and without change (27.5% and 28.2%). Carrying a weapon almost doubled (4.3% to 7.1%). Less than 65% reported classes for violence prevention.

**Conclusions:**

The GSHS-Lara shows that violence is an important public health problem that needs to be addressed by the community and its authorities.

## Introduction

Youth violence, an aggressive and hostile behavior amongst youth, is one of the most visible forms of violence in society and a major public health problem worldwide [1]. The World Report on Violence and Health-2000 indicated that fatal forms of violence (i.e. any form of violence and injuries that result in homicide) takes the lives of 545 people aged 10 to 29 years as a result of interpersonal violence each day [2]. Those who survived suffered a range of negative changes in their growth and development including increasing risk for physical, mental, social and intellectual problems [3]. However, homicide represents only the 'tip of the iceberg' as far as violence is concerned. The World Health Organization (WHO) estimated that for every youth homicide there could be 40 youth victims of non-fatal violence receiving hospital treatment [2]. However, the real magnitude of the problem could not be calculated as the number of youth victims of violence that never reach the hospital is unknown. Violence amongst students attending school is a particularly sensitive issue because it challenges the established social concept that schools are safe places for students and teachers; this issue is gaining public notoriety as the mass media expose the situation of violence in schools. Hopefully it will force communities and governments to take action to solve the problem; otherwise the situation could cause severe social damage [4].

In societies where citizens have to deal with violence and crime on a daily basis, public attention is focused on violent crimes; therefore, the common forms of violence found at schools such as non-physical violence (for example, verbal harassment), physical attacks, fights, and certain threats using weapons are considered 'normal' facts of life. Indeed, violence in Venezuela is considered a main health and social problem. In 2008, there were 16,049 murder cases, that is 54 per 100,000 inhabitants, which is one of the highest rates in the Americas. Additionally, non official reports indicate that in only 9% of murder cases a suspect is detained, and the conviction rate is even lower [5]. However, they are important because they disrupt the educational process and they have the possibility of promoting criminal behavior [3].

As in other types of behavior, the profile of violence amongst adolescents is constantly changing. Therefore, valid, current and accessible information to deal with this problem is a constant need for health promotion and violence prevention. The World Health Organization (WHO) has advocated for an international surveillance system that produces comparable data regarding violence by and against youth [1]. These data are critical for development, implementation and evaluation of public policies and programs intended to reduce such violence [6-10]. In this article, we report trends of common violence markers in two different generations of Venezuelan students of the 7th to 9th grade using data from the Global School-based Student Health Survey (GSHS) in The State of Lara (Venezuela), that has a population of 1,554,415 inhabitants, 33% below the age of 15, 84% living in urban areas, with a density of 78.6 people/km^2 ^[11].

## Methods

The GSHS is a school-based international health surveillance system, designed to observe selected aspects of health and health determinants among youth, following a standard protocol [9]. The GSHS is an initiative of the World Health Organization (WHO) with the technical assistance of the Centers for Disease Control and Prevention (CDC) of the United States of America (USA), in a global alliance with governmental and non-governmental organizations [10]. The ten-year-old GSHS follows the methodology of the Global Youth Tobacco Survey (GYTS) [7,8,12]. The methodology of the Venezuelan Lara State GSHS (GSHS-Lara) has been described in detail elsewhere [13-15]. In summary, the sampled population is composed of students enrolled in 7th, 8th, and 9th grade during the school years of 2003-2004 (GSHS-Lara 2004) and 2007-2008 (GSHS-Lara 2008). The sample design had two stages: the first consisted of sampling schools with the probability of selection proportional to the school enrollment size and the second stage consisted of randomly selecting classes from the 7th, 8th and 9th grades. Within each selected class, all students were invited to participate. The protocol of the GSHS-Lara was approved by the Ethical Committee of ASCARDIO. The authorities of each school provided permission to administer GSHS-Lara. The privacy of the students and their free anonymous participation were assured. The questionnaire has a core of questions common to all places where GSHS is applied as well as questions to address specific needs of Lara State. Both are presented in modules to address health areas: sexual behavior, tobacco use, nutrition, violence, mental health, physical activity and hygiene, among others. The WHO and CDC developed the core questions. The specific questions for the students in Lara State were developed by ASCARDIO. The Spanish version of the survey core was validated through: (a) review by experts; (b) pilot test and (c) student focus groups. Consistency among versions of the questionnaire (Spanish and English) was assessed by comparing different versions translated by different independent translators. GSHS was administered by trained personnel of the Cardiovascular Diseases Program of Lara State, Venezuela, Ministry of Health (CVDP-MH) and ASCARDIO.

The WHO defines violence as: "The intentional use of physical force or power, threatened or actual, against oneself, another person, or against a group or community that either results in or has a high likelihood of resulting in injury, death, psychological harm, maldevelopment or deprivation" [16]. Built upon this definition, the GSHS assessed the following violence indicators as follows: (A) absenteeism of the students due to feeling unsafe at school or on the way to or from school by percentage of students who did not attend classes one or more times during the last 12 months, (B) robbery by percentage of students who were robbed one or more times inside their school in the last 12 months and percentage of students who were robbed one or more times outside their school in the last 12 months; (C) bullying by percentage of students who were bullied one or more days during the past 30 days plus percentage of bullied students who were bullied most often by being hit, kicked, pushed, shoved around or locked indoors; (D) physical fights by percentage of students who were in a physical fight one or more times during the past 12 months and the percentage of students who were physically attacked one or more times during the past 12 months; (E) the use of weapons by percentage of students who carried a weapon, such as a gun, knife, or club on one or more days during the past 30 days. In addition, we analyzed the exposure to formal lectures focused on the prevention of violence [10]. These indicators were presented as prevalence per 100 responders (P%) with the corresponding 95% confidence interval (CI95%). All data were calculated using the C Sample statistical program within Epi-Info version 3.5.1 from CDC. The weighting factor was applied to each student record to adjust for non-responses and for the varying probabilities of selection. Prevalence estimates between two time periods were considered to be statistically significantly different from each other (StatSig) at the 0.05 level (2-sided), if their 95% confidence intervals do not overlap.

## Results

Table 1 presents both the weighted prevalence per 100 students (P%) and its CI95%. The prevalence of students who did not attend classes (absenteeism) 1 or more times due to feeling unsafe at school or on the way to or from school during the last 12 months saw a nearly twofold increase for males (13.1% (2004) to 25.2% (2008)) and females (8.8% (2004) to 16.7% (2008)).

**Table 1:** Prevalence of Selected Indicators for Violence among Students of the 7th to 9th Grade in Lara State, Venezuela.

	Total		Males		Females	
	2004	2008	2004	2008	2004	2008
	n = 2070	n = 870	n = 860	n = 383	n = 1210	n = 411
Indicators	P(%)	P(%)	P(%)	P(%)	P(%)	P(%)
	IC 95%	IC 95%	IC 95%	IC 95%	IC 95%	IC 95%
**Absenteeism (a)**	10.8	20.8	13.1	25.2	8.8	16.7
	8.7-13.0	13.1-28.5	10.7-15.6	15.2-35.1	6.0-11.6	9.0-24.4
**Robe (b)**	14.2	14.8	16.0	17.7	12.7	12.2
A - Out of school	11.8-16.6	10.6-19.0	13.0-19.0	12.4-23.0	10.4-14.9	7.7-16.7
B - In-school	21.7	22.0	20.9	26.9	22.5	17.1
	19.5-23.8	14.6-29.5	18.1-23.7	16.6-37.3	19.5-25.6	11.2-23.0
**Bullying (c)**	33.4	43.6	35.6	46.2	31.4	41.1
A - Per ≥ 1 days	30.3-36.5	35.8-51.3	31.9-39.3	36.3-56.1	26.7-36.0	33.5-48.7
B - Hit, kicked, pushed, shoved around, or locked indoors	18.5	14.3	27.8	17.9	8.3	11.0
	12.8-24.3	9.7-18.8	20.1-35.6	10.8-25.1	5.5-11.1	5.7-16.3
**Physical attack (d)**	NA	21.5	NA	28.2	NA	15.2
A - Physically attacked	NA	15.8-27.2	NA	20.6-35.7	NA	9.1-21.4
B - Physical fight	27.5	28.2	44.9	43.1	12.0	14.7
	22.3-32.7	23.7-32.7	40.5-49.2	36.1-50.1	10.3-13.8	10.8-18.5
**Weapon (e)**	4.3	7.1	7.2	10.9	1.8	3.6
Carrying any weapon	3.1-5.6	3.7-10.5	5.4-8.9	6.1-15.8	1.0-2.6	0.9-6.2

The Global School-based Student Health Survey (GSHS), 2004 and 2008.

**2004 and 2008**: corresponding to the academic school years 2003-2004 and 2007-2008, respectively; **P(%): **prevalence per 100 participants; **IC95%: **95% Confidence Interval. **NA **Not available

Indicators for violence

**(a) Absenteeism (in the last 12 months): **students who did not attend classes one or more days due to feeling unsafe at their school or on their way to or from school.

**(b) Robbery (in the last 12 months): A-**Students who were robbed one or more times outside their school. **B-**Students who were robbed one or more times inside their school.

**(c) Bullying (during the past 30 days): A-**Students who were bullied on one or more days. **B-**Students who were bullied most often by being hit, kicked, pushed, shoved around, or locked indoors.

**(d) Physical attack (in the last 12 months): A-**Students who were physically attacked one or more times. **B-**Students who were in a physical fight one or more times.

**(e) Carrying a weapon (during the past 30 days): **Students who carried a weapon, such as a gun, knife, or club on one or more days.

Considering both sexes, students are about 7% more likely to be robbed inside than outside school. However, the P% of "being robbed inside and outside school" remained unchanged (2004 vs 2008): inside (14.2% vs 14.8%) and outside (21.7% vs 22.0%).

The prevalence of being bullied increased (2004 vs 2008): for males from 35.6% to 46.2% and for females from 31.4% to 41.1%. Bullying associated with physical violence decreased (between 2004 and 2008) only among males and increased among females.

The prevalence of being physically attacked in the previous 12 months, a new indicator in GSHS-Lara 2008, was reported by 28% of males and 15% of females. Almost 5 out of every 10 males were involved in a physical fight (44.9% vs. 43.1%) while the figures were lower for females (12.0% vs. 14.7%). The prevalence of students carrying weapons increased (2004 vs. 2008): for males 7.2% vs. 10.9% and for females 1.8% vs. 3.6%, but without StatSig for females. Table 2 shows that less than 7 out of every 10 responders indicated having had lectures on how to avoid physical fights and violence; less than 5 out of every 10 students reported receiving lectures on how to avoid being bullied, or lectures on what to do if someone tries to force you to have sexual intercourse.

**Table 2:** Prevalence of Selected Indicators for Violence Prevention at School among Students of the 7th to 9th Grade in Lara State, Venezuela.

	Total		Males		Females	
	2004	2008	2004	2008	2004	2008
	n = 2070	n = 870	n = 860	n = 383	n = 1210	n = 411
Indicators	P(%)	P(%)	P(%)	P(%)	P(%)	P(%)
	IC 95%	IC 95%	IC 95%	IC 95%	IC 95%	IC 95%
**Avoiding physical violence (a)**	56.2	64.5	56.1	64.3	56.2	64.8
	52.2-60.1	59.5-69.4	50.9-61.4	57.0-71.6	50.9-61.4	59.3-70.2
**Avoiding violence from bullies (b)**	46.0	43.3	47.6	41.3	44.1	44.8
	41.3-50.8	38.5-48.0	42.8-52.5	36.0-46.6	37.5-50.7	38.8-50.8
**Avoiding sexual violence (c)**	29.5	40.1	27.1	36.2	31.7	43.1
	27.3-31.6	33.7-46.6	23.7-30.6	29.5-42.9	28.7-34.6	36.3-49-8

The Global School-based Student Health Survey (GSHS), 2004 and 2008

**2004 and 2008**: corresponding to the academic school years 2003-2004 and 2007-2008, respectively; **P(%): **prevalence per 100 participants; **IC95%: **95% Confidence Interval. **NA **Not available

Indicators for violence prevention

**(a) Avoiding physical violence: **Students who reported having been taught "how to avoid physical fights and violence".

**(b) Avoiding violence from bullies: **Students who reported having been taught "how to avoid being bullied".

**(c) Avoiding sexual violence: **Students who reported having been taught "what to do if someone is trying to force you to have sexual intercourse".

## Discussion

There are limitations of using cross-sectional data to describe changes over time. Time trends obtained from cross-sectional data have a different meaning than time trends obtained from cohort data. Cohort studies follow up an initial study population over time, and therefore the time trends refer to changes in the individuals over time, and are best suited for risk analysis. Cross-sectional studies use a new representative sample of the population each time, so the time trends refer to changes in the population over time, and are appropriate for burden analysis. A surveillance system seeks information on the same issues over an extended period of time. Therefore, data can be useful for determining adequate strategies and prevention, as is the case in this article, that presents data from a surveillance system.

In the past, to face violence in schools by removing students suspected of committing violent acts and asking the judiciary system to take the punitive leadership has been an unsuccessful policy to stop and/or decrease school violence [17]. Most recently, a holistic approach to school violence looks promising because not only does it include the criminal act committed by minors, it also takes into account the cultural, social, economic and environmental factors that promote violence [18,19]. The superiority of the holistic approach has been shown by comparing different typologies of school-based violence prevention strategies. Interventions that use psychosocial and psycho-educational programs or multiple strategies with key stakeholder groups working to reduce aggression reveal strong evidence for prevention. Conversely, the use of standard strategies for the entire school or school district such as security apparatus and policies, peer-led programs, discipline policies and rules, threat assessment and crisis response have shown poor or minimal evidence of violence prevention most of the time [20]. More recently, those programs that link the interests of families and teachers to build social skills among students from earlier grades (≥ 5th) and target multilevel approaches to high risk populations with abundance of violence among adolescents have resulted in a substantial reduction of school dropout, fewer delinquency reports, fewer arrests by age 19, and increasing social skills among adolescents [21]. Other validated violence prevention programs are listed at the end of this article.

To accomplish a holistic, preventative, school-based approach against violence, it is necessary to understand the epidemiological profile of violence in the school based on comparative evidence as provided by the GSHS [1-3,22]. Data from the first GSHS is currently available for some Latin American countries, a region where school violence among youth is escalating [23-30].

Absenteeism is an unspecific but useful proxy indicator of violence in schools used in many studies [31-37]. However, in the Americas, only a handful of countries allow access to official data on school absenteeism. The GSHS-Lara shows that absenteeism is a growing problem. The prevalence of students who avoid going to school due to feeling unsafe at their school or on their way to or from school doubled from 1 in 9 students (2004) to 1 in 5 students (2008), figures being higher for males (13.1% 2004 vs. 25.2% 2008) than for females (8.8% 2004 vs. 16.7% 2008). This makes GSHS-Lara 2008 the third highest prevalence after the 28.4% of GSHS-Santiago, metropolitan area 2005 (Chile) and 21.25% of GSHS-Quito 2007 (Ecuador).

Being robbed inside and outside of school can be a contributor to absenteeism among students because it produces feelings of futility, inadequacy, and loss in the victim [38]. In the GSHS-Lara, students were robbed more frequently inside the school than outside of the school: 21.7% (2004) and 22% (2008) in the school, and 14.2% (2004) and 14.8% (2008) elsewhere, respectively.

Bullying, physical fighting, and carrying a weapon are important indicators of youth violence, which not only contribute to student absenteeism but can also lead to more severe forms of violence [1]. GSHS-Lara showed that the P% of students who suffered from bullying increased for both genders, i.e. in males from 35.6% (2004) to 46.2% (2008) and in females from 31.4% (2004) to 41.1% (2008); figures close to 1 in 2, which is comparable with the GSHS-Santiago, Metropolitan Area 2005 (Chile); followed by the USA and GSHS-Bogotá 2007 (Colombia), close to 1 in 3; with the third spot for the GSHS Argentina-2007 and GSHS-Quito 2007 (Ecuador), close to 1 in 4 [25-27,30,39].

Bullying is often considered an inevitable part of growing up, a kind of "small and subtle violence" if compared with those cases of "high violence" associated with crime [3]. Currently bullying is known to be one of the most prevalent dire experiences that students have to endure at school, as it is associated with physical aggression, verbal harassment, and psychological manipulation leading to difficulty in internalizing moral values, escalating anger, school absenteeism, poor academic performance, mood disorders, humiliation, abuse of substances and eating disorders, to name but a few [2]. The GSHS-Lara showed that at least 1 in 7 male students was a victim of bullying associated with physical attacks (18.5% in 2004 vs. 14.3% in 2008) and unfortunately, there is an increasing prevalence for females (8.3% in 2004 vs. 11.0% in 2008), as you can see in Table 1.

Physical fighting is very common among school-age children in many parts of the world [1]. The GSHS-Lara 2008 showed that 28.2% of males and 15.2% of females had been physically attacked. These data place Venezuela in third place compared with GSHS reports from other Latin American countries such as Argentina and Chile [26-28,30]. The prevalence of active participation in physical fighting is high and remains unchanged over time: males 44.9% (2004) vs. 43.1% (2008) and females 12.0% (2004) vs. 14.7% (2008). These results are consistent with international reports indicating that one-third of the male students have been involved in fighting [1]. Compared with other Latino countries, Venezuela is in third place after Chile (close to 1 in 2) and the rest of GSHS in Latin American such as Argentina, Colombia and Ecuador (close to 1 in 3) [25,26,29,30]. Data from GSHS-Lara show that the number of students carrying a weapon increased for both genders to 1 in 9 males and 1 in 28 females in 2008, figures that are close to those reported in Cape Town, South Africa for males (1 in 10); but below those reported for the USA (1 in 4 males and 1 in 15 females in 9th to 12th grade) and the Netherlands (1 in 8 for both sexes) and Scotland (1 in 3, also for both sexes) [1]. The GSHS-Lara did not explore where weapons had been obtained; other surveys showed that youth often have access to guns at home, where it is common for parents to have positive perceptions of their children's understanding and behavior towards guns, i.e. 75% of parents thought their children [ages 4 to 12 years] could tell the difference between a toy gun and a real gun, and 53% said they could trust their children not to touch loaded guns. However, the reality indicates that the possession of a gun may facilitate a homicide when adolescents are involved in physical conflicts [1-3]. Finally, despite the profile of violence shown by the GSHS-Lara, students did not report any improvement in exposure to violence prevention lectures at school.

## Conclusions

Data from the GSHS-Lara 2004 and 2008 give evidence for the urgency of implementing programs with proven strong evidence of violence prevention among adolescents. There is also an urgent need for policies that approach the increasing violence at school by taking into account cultural, social, economic, and environmental factors involved. Efforts for school-based health promotion and prevention programs must be continuous and supported by a permanent surveillance system.

## Competing interests

The authors declare that they have no competing interests.

## Authors' contributions

RG conceived of the study, and participated in its design and coordination, designed the data analysis process and participated in the sequence alignment and drafted the manuscript. EP carried out literature research, participates in the data analysis process and in the sequence alignment and drafted the manuscript. BE carried out literature research, participates in the data analysis process and in the sequence alignment and drafted the manuscript. JE carried out literature research, participates in the drafted of the manuscript. All authors read and approved the final manuscript

## References

[B1] ITS Global Consultation on Violence and Health Violence: a public health priority 1996 Geneva, World Health Organization (document WHO/EHA/SPI.POA.2)

[B2] Krug EG Dahlberg LL Merch JA Zwi AB Lozano R World report on violence and health 2002 Geneva (Switzerland): World Health Organization (WHO) 1 44

[B3] Pratt HD Greydanus DE Violence: concepts of its impact on children and youth Pediatr Clin N Am 2003 50 963 1003 10.1016/S0031-3955(03)00083-X 14558678

[B4] Miller AK Chandler K Violence in U.S. Public Schools: 2000 School Survey on Crime and Safety Statistical Analysis Report. Supported by U.S. Department of Education, Institute of Education Sciences and National Center for Education and Statistics, October 2003 2005

[B5] Informe 2008 de Violencia en Venezuela, Observatorio Venezolano de Violencia http://www.observatoriodeviolencia.org.ve/site/

[B6] Heymann DL Rodier GR Special Issue: Global Surveillance of Communicable Diseases Emerging Infectious Diseases 1998 4 3 World Health Organization, Geneva, Switzerland http://www.cdc.gov/ncidod/eid/vol4no3/heymann.htm Last checked on 8/10/2009 10.3201/eid0403.980305 PMC2640271 9716946

[B7] The Global Youth Tobacco Survey Collaborative Group Tobacco control Tobacco use among youth: a cross-country comparison 2001 11 252 270 10.1136/tc.11.3.252 PMC1759013 12198280

[B8] Warren C Riley L Asma S Eriksen M Green L Blanton C Loo C Batchlor S Yach D Tobacco use by youth: a surveillance report from the Global Youth Tobacco Survey Project 2000 78 Bulletin of the World Health Organization 868 876 PMC2560802 10994259

[B9] Choi B Corber S McQueen D Bonita R Zevallos J Douglas K Barceló K Gonzalez M Robles S Stachenko S Hall M Champagne B Lindner C Salazar L Granero R Soto L Lum W Torres R Warren C Mokdad A Enhancing regional capacity in chronic disease surveillance in the Americas Rev Panam Salud Publica/Pan Am J Public Health 2005 17 131 141 10.1590/s1020-49892005000200012 15826391

[B10] United States Department of Health and Human Services, Centers for Disease Control and Prevention, National Center for Chronic Disease Prevention and Health Promotion, Division of Adolescent and School Health. Questionnaire-CDC Global School-based Student Health Survey (GSHS) Rationale. Available from: URL: Global School-based Student Health Survey 2005 CDC http://www.cdc.gov/GSHS

[B11] Gobierno Bolivariano de Venezuela, FUDECO Resumen de datos socio demográficos Estado Lara http://www.fudeco.gob.ve/estados.php Last checked on 10/09/2011

[B12] Frieden T CDC in the News: Global Tobacco Surveillance System Serves as Worldwide Standard 2009 http://www.cdc.gov/news/2009/07/gtss/Last checked on 08/11/2009

[B13] Granero R Poni E Sánchez Z Patrones de actividad física durante el tiempo de ocio entre los estudiantes del séptimo al noveno grado en el Estado Lara, Venezuela Avances Cardiol 2007 27 160 167

[B14] Granero R Poni E Sánchez Z Sexuality among 7th, 8th, and 9th grade students in the State of Lara, Venezuela. The Global School Health Survey, 2003-2004 PR Health Sci J 2007 26 213 219 18035813

[B15] Granero R Poni E Poni C Suicidal ideation among students of the 7th, 8th, and 9th grades in the State of Lara, Venezuela: The Global School Health Survey PR Health Sci J 2008 27 337 342 19069360

[B16] Krug EG Dahlberg LL Merch JA Zwi AB Lozano R World report on violence and health 2002 Geneva (Switzerland): World Health Organization (WHO) 5 http://whqlibdoc.who.int/publications/2002/9241545615_chap1_eng.pdf

[B17] Gajardo M Desde distintas realidades nacionales: Pistas para abordar la violencia escolar. En: Formas & Reformas de la Educación - Serie Prevención de la Violencia Escolar. PREAL Mayo/2005/Año 3 - N° 6 Chile http://www.preal.org Last checked on 9/10/2009

[B18] International Juvenile Justice Observatory programs http://www.oijj.org Last checked on 9/10/2009

[B19] Cámara L Assis SG Oliveira RVC Violência Escolar e Auto-Estima de Adolescentes Cadernos de Pesquisa 2006 36 127 35 50 10.1590/S0100-15742006000100003

[B20] Greene MB Reducing Violence and Aggression in Schools TRAUMA, VIOLENCE & ABUSE 2005 6 3 236 253 10.1177/1524838005277406 16237157

[B21] Eisenbraun KD Violence in schools: Prevalence, prediction, and prevention Aggression & Violent Behavior 2007 12 4 459 469 10.1016/j.avb.2006.09.008

[B22] Mytton JA DiGuiseppi C Gough DA Taylor RS Logan S School-Based Violence Prevention Programs: Systematic Review of Secondary Prevention Trials Arch Peds Adol Med 2002 156 8 752 762 10.1001/archpedi.156.8.752 12144364

[B23] Rodgers D Latin America and Caribbean Region Sustainable Development Working Paper No. 4. Urban Peace Program Series: Youth Gangs and Violence in Latin America and the Caribbean: A Literature Survey, August 1999 The World Bank - Latin America and Caribbean Regional Office - Environmentally and Socially Sustainable Development SMU

[B24] Briceño-León R Zubillaga V Violence and Globalization in Latin America Current Sociology 2002 50 1 19 37 10.1177/0011392102050001003

[B25] Linetzky B Encuesta Mundial de Salud Escolar 2007 en Argentina http://www.cdc.gov/GSHS

[B26] Global School-based Student Health Survey 2005 Fact Sheet in Chile (Metropolitan Mun., without Intervention) http://www.cdc.gov/GSHS

[B27] Global School-based Student Health Survey 2007 Fact Sheet, in Colombia (Bogotá, official schools) http://www.cdc.gov/GSHS

[B28] Global School-based Student Health Survey 2007 Fact Sheet, in Colombia (Bogotá, private schools) http://www.cdc.gov/GSHS

[B29] Global School-based Student Health Survey 2007 Fact Sheet, in Colombia (Bogotá) http://www.cdc.gov/GSHS

[B30] Global School-based Student Health Survey 2007 Fact Sheet, in Ecuador (Quito) http://www.cdc.gov/GSHS

[B31] Kearney CA An Interdisciplinary Modelo f School Absenteeism in Youth to Inform Professional Practice and Public Policy Edu Psych Rev 2008 20 3 257 282 10.1007/s10648-008-9078-3

[B32] McCray E It's 10 a.m.: Do you know where your children are? The Persisting Issue of School Truancy Intervention in School and Clinic 2006 42 30 33 10.1177/10534512060420010501

[B33] Hansen C Sanders S Massaro S Last C Predictors of Severity of Absenteeism in Children with the Realm of Juvenile Justice Family Relations 2006 55 190 199 10.1111/j.1741-3729.2006.00369.x

[B34] Reimer M Smink J Information About the School Dropout Issue Selected Facts 6 Statistics A publication of the National Dropout Prevention Center/Network 2005

[B35] Zhang M Time to Change the Truancy Laws? Compulsory Education: Its Origin and Modern Dilemma Napce 2002 24 27 34

[B36] Garry E Truancy: First Step to Lifetime of Problems Office of Juvenile Justice and Delinquency Prevention 1996

[B37] Kearney CA School absenteeism and school refusal behavior in youth: A contemporary review Clin Psychol Rev 2008 28 3 451 471 10.1016/j.cpr.2007.07.012 17720288

[B38] Lucile Packard Foundation for Children's Health-Child Health and Related News: #1 Bullying Behaviors Among US Youth: Prevalence and Association With Psychosocial Adjustment 2001 10.1001/jama.285.16.2094 PMC2435211 11311098

[B39] U.S. Department of Justice-Office of Justice Programs Bureau of Justice Statistics. Victim characteristics: Violent crimes, summary findings 2006 http://bjs.ojp.usdoj.gov/index.cfm?ty=pbdetail&iid=765 Last checked on 10/09/2011

